# Graphene Nanoplatelets as Novel Reinforcement Filler in Poly(lactic acid)/Epoxidized Palm Oil Green Nanocomposites: Mechanical Properties

**DOI:** 10.3390/ijms130910920

**Published:** 2012-08-30

**Authors:** Buong Woei Chieng, Nor Azowa Ibrahim, Wan Md Zin Wan Yunus, Mohd Zobir Hussein, V. S. Giita Silverajah

**Affiliations:** 1 Department of Chemistry, Faculty of Science, Universiti Putra Malaysia, UPM Serdang, Selangor 43400, Malaysia; E-Mails: mzobir@science.upm.edu.my (M.Z.H.); vsgiita@gmail.com (V.S.G.S.); 2 Chemistry Department, Center for Defence Foundation Studies, National Defence University of Malaysia, Kuala Lumpur 57000, Malaysia; E-Mail: wanmdzin@upnm.edu.my

**Keywords:** graphene nanoplatelets, biodegradable plastic, mechanical properties

## Abstract

Graphene nanoplatelet (xGnP) was investigated as a novel reinforcement filler in mechanical properties for poly(lactic acid) (PLA)/epoxidized palm oil (EPO) blend. PLA/EPO/xGnP green nanocomposites were successfully prepared by melt blending method. PLA/EPO reinforced with xGnP resulted in an increase of up to 26.5% and 60.6% in the tensile strength and elongation at break of the nanocomposites respectively, compared to PLA/EPO blend. XRD pattern showed the presence of peak around 26.5° in PLA/EPO nanocomposites which corresponds to characteristic peak of graphene nanoplatelets. However, incorporation of xGnP has no effect on the flexural strength and modulus. Impact strength of PLA/5 wt% EPO improved by 73.6% with the presence of 0.5 wt% xGnP loading. Mechanical properties of PLA were greatly improved by the addition of a small amount of graphene nanoplatelets (<1 wt%).

## 1. Introduction

Recently, renewable bio-based polymeric materials have been widely developed and have gained much attention in the scientific community due to concern regarding the global environment and for use as an alternative to the petroleum-based polymeric materials which are commercially used [1]. Bio-based polymeric materials can be used as a substitute to overcome the problems of non-biodegradable and synthetic resin materials, which are difficult to deal with and give rise to relatively large quantities of daily generated waste.

**Figure 1:**
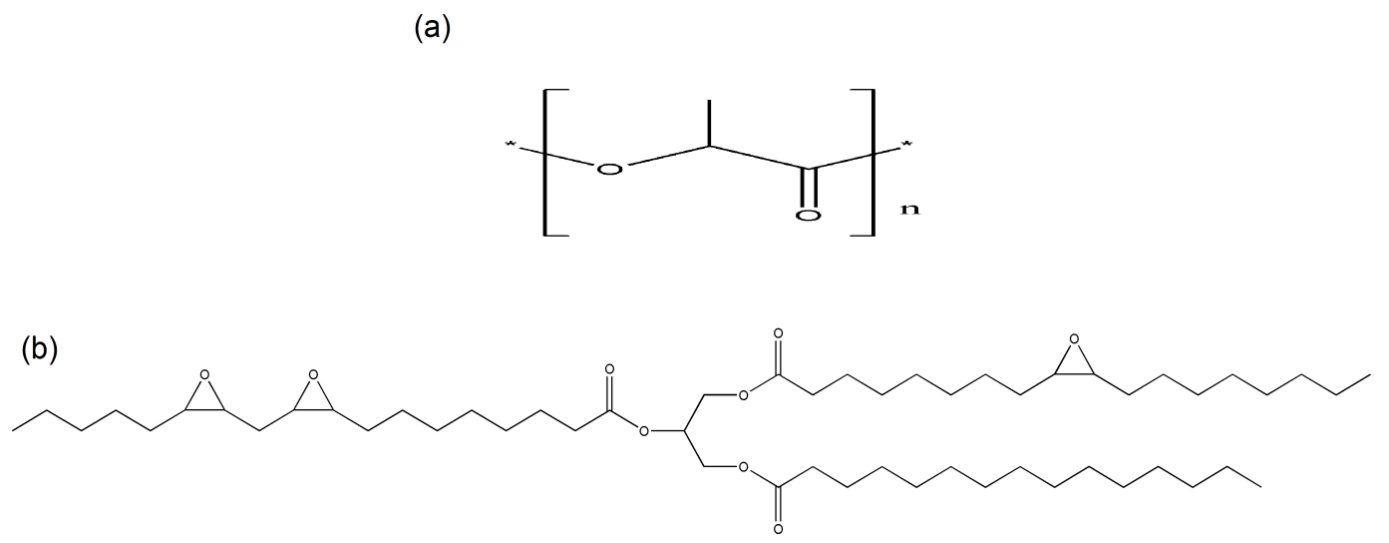
Chemical structures of (**a**) poly(lactic acid) (PLA) and (**b**) epoxidized palm oil (EPO).

Biodegradable polymer products based on renewable agricultural feedstock can form the basis for a portfolio of eco-efficient products, which can compete with currently dominating products based on petroleum feedstock. Poly(lactic acid) (PLA), cellulosic plastics, polyhydroxy alkonate, thermoplastic starch, vegetable oils, and palm oils are examples of renewable resources based biopolymers. PLA can be obtained from renewable resources by a fermentation process using sugar from corn, either by ring-opening polymerization or condensation polymerization [2]. PLA is linear aliphatic thermoplastic polyester (Figure 1a) that is readily biodegradable via enzyme action [3]. However, PLA causes drawbacks for some applications, owing to its brittleness, mechanical, thermal, barrier, and flame retardant properties [4].

Attempts have been made to improve flexibility by blending PLA with other polymers such as poly(ɛ-caprolactone) (PCL), poly(butylene succinate) (PBS) [5], poly(ether urethane), and poly(butylene adipate-co-terephthalate) (PBAT) [6]. However, all the mentioned polymers are petrochemically-derived polymers. Some efforts to modify PLA have also been performed for example with tributyl citrate [7], oligomeric malonate esteramides [8], polyglycerol esters [9], and poly(1,3-butylene adipate) [10] to improve its elongation at break. Epoxidized palm oils (EPO) is one of the potential candidates as a substitution for the petrochemical-based plasticizers, as the EPO are natural-based, renewable, biodegradable, low toxicity, cost effective and capable to minimize the emission of carbon dioxide gas which gives rise to global warming phenomenon [11]. Furthermore, EPO may simultaneously provide an intrinsic thermal stabilization effect due to the presence of oxirane rings (Figure 1b) [12].

Concerning mechanical aspects, properties of PLA can be improved by addition of nano-sized filler. Various nano-reinforcement filler, such as layered silicate clay [13], carbon nanotubes [14] and layered double hydroxide [15] are being developed and extensively studied. However, the discovery of new nano-material graphene by Andre Geim in year 2004 [16], excited worldwide interest among researchers. Graphene, which is fabricated from natural graphite can also be used as a potential alternative nano-reinforcement to both clay and carbon nanotubes. Graphene combines layered structure of clays with superior mechanical and thermal properties of carbon nanotubes, which can provide excellent functional properties enhancements [17]. Since graphite is the stiffest material found in nature having a modulus several times higher than clay, combined with its excellent strength, electrical and thermal conductivity, it ought to have similar properties as carbon-based nanomaterials [18]. Furthermore, graphene is much cheaper than either single-walled carbon nanotubes or multi-walled carbon nanotubes. It is well known that the graphene/polymer nanocomposites generally have increased mechanical strength compared to pristine polymers. There are many studies on graphene composites based on a range of polymers available in literature [19–21], e.g., polycarbonate (PC), poly(ethylene-2,6-naphthalate (PEN), poly(caprolactone) (PCL), Nylon-6, poly(methyl methacrylate) (PMMA), poly(vinylidene fluoride) (PVDF), epoxy, polyurethanes, poly(vinyl alcohol), polyamide 6 (PA6), polystyrene (PS) and poly(lactic acid) [22]. Effective mechanical reinforcement of polymeric material using very low amounts of graphene has been reported by several authors. Pinto and co-workers, improved the tensile strength of PLA film by 15% and Young’s modulus by 85% incorporating 0.4 wt% graphene nanoplatelets [23]. An increase of about 75% in Young’s modulus and 74% in yield strength of polypropylene was achieved at 0.42 vol% graphene oxide loading by Song and co-workers [24]. Cao and co-workers increased Young’s modulus of PLA by 18% with the addition of only 0.2 wt% of reduced graphene oxide [25].

In this study, graphene nanoplatelet (xGnP) was investigated as novel nano-reinforcement filler in PLA/EPO blend by melt blending techniques. Significant improvements in the tensile strength and elongation at break were observed when xGnP was incorporated as reinforcement filler into PLA/EPO blend.

## 2. Results and Discussion

### 2.1. X-ray Diffraction (XRD)

**Figure 2:**
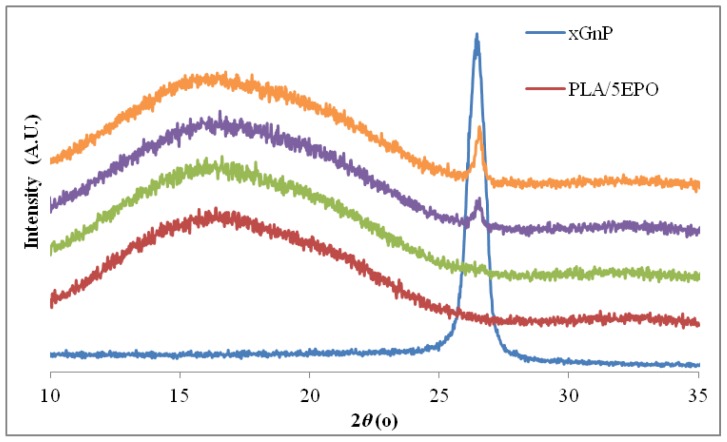
XRD patterns of graphene, PLA/EPO and PLA/EPO nanocomposites.

XRD is an effective method to determine whether xGnP exist as individual graphene sheets in the nanocomposites. Figure 2 shows the XRD patterns of as-received xGnP, PLA/EPO and selected PLA/EPO composite with various xGnP loading. The xGnP exhibits an intense peak at 2*θ* value of ~26.4°, assigned to the stacking of the single graphene layers at a distance of 0.34 nm [26]. There is no other peak observed for xGnP. On the other hand, the XRD patterns of PLA/5EPO blend and PLA/5EPO with various xGnP loadings exhibit an initial broad characteristic peak of PLA matrix at 2*θ* = ~16°. Notice that, there was no xGnP’s peak observed for PLA/5EPO/0.1 wt% which may be due to the low amount of ordered layer structure of xGnP. The disappearance of peak may also be due to the exfoliation and random distribution of the platelets within the polymer matrices at low loading of xGnP.

A small peak around 26.5° which corresponds to characteristic peak of xGnP start emerges at 0.3 wt% xGnP laoding in PLA/5EPO nanocomposites, showing that the graphene layer is unable to disperse or completely separate and some sheets are still present in stacks form. The intensity of this peak increases as the xGnP loading increases. The increased intensity recorded at higher xGnP loading could be attributed to the higher number of graphene layers organized in stacks. Similar results have been previously reported and the peak observed of reduced intensity was associated to a lower number of graphene stacks [27].

### 2.2. Brunauer-Emmett-Teller (BET)

**Figure 3:**
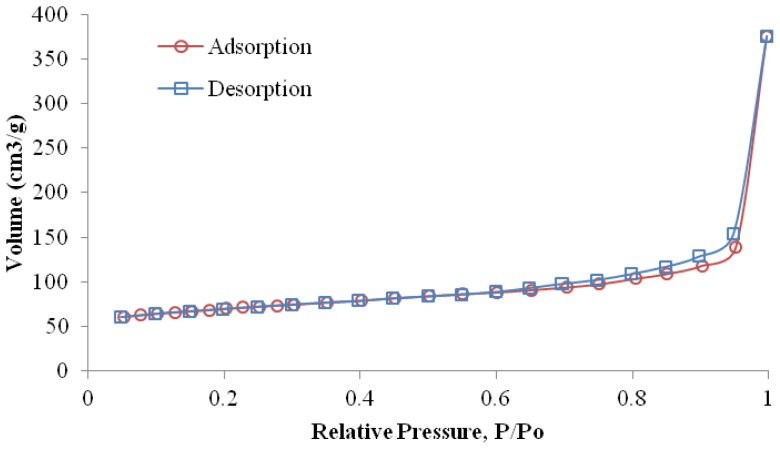
Nitrogen adsorption/desorption isotherms of graphene nanoplatelets.

The N_2_ adsorption-desorption isotherm of graphene nanoplatelets is shown in Figure 3. The BET specific surface area of graphene nanoplatelets is about 226.8 m^2^/g compared to the graphite powder which is about 8.5 m^2^/g (the data was not shown). This indicates that the average particle size of graphite has been decreased. However, the specific surface area (226.8 m^2^/g) of the xGnP is smaller than the theoretical specific surface area of single-layer graphene sheets (2620 m^2^/g). A hysteresis loop in the nitrogen adsorption/desorption isotherms of graphene was also observed. The hysteresis loop resembles type H3 in IUPAC (International Union of Pure and Applied Chemistry) classification, resulting from slit-shaped pores between parallel layers [28]. xGnP are stacks of a few graphene sheets and the porosity might arise from N_2_ gases penetration within the inter-sheet slits. The total pore volume of xGnP is as high as 0.488 cm^3^/g, but the micropore volume is only 0.011 cm^3^/g, revealing a very low micropore content. The average pore diameter is about 1.713 nm calculated by the BET model.

### 2.3. Tensile Properties

**Figure 4:**
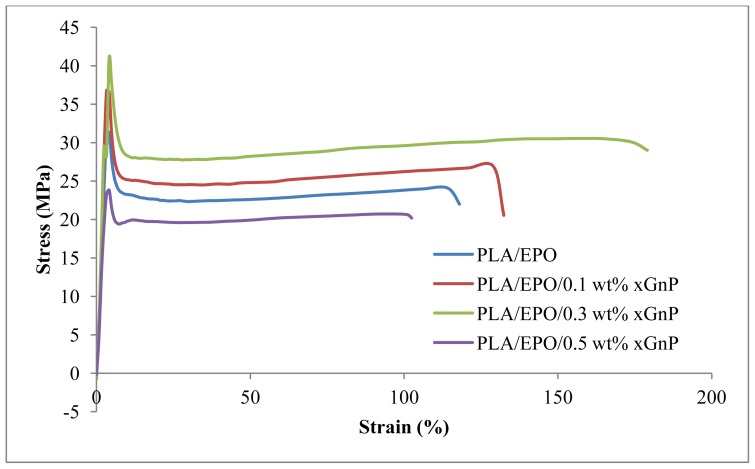
Stress-strain curves for PLA/EPO and PLA/EPO/xGnP nanocomposites.

**Figure 5:**
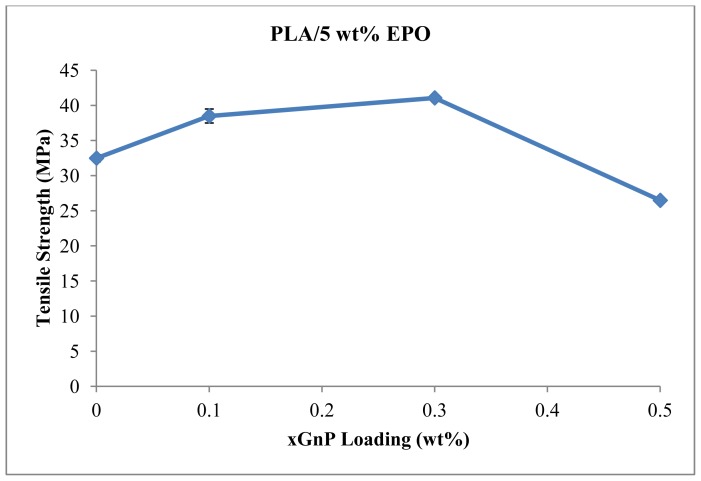
Tensile strength of PLA/5EPO with various xGnP loadings.

**Figure 6:**
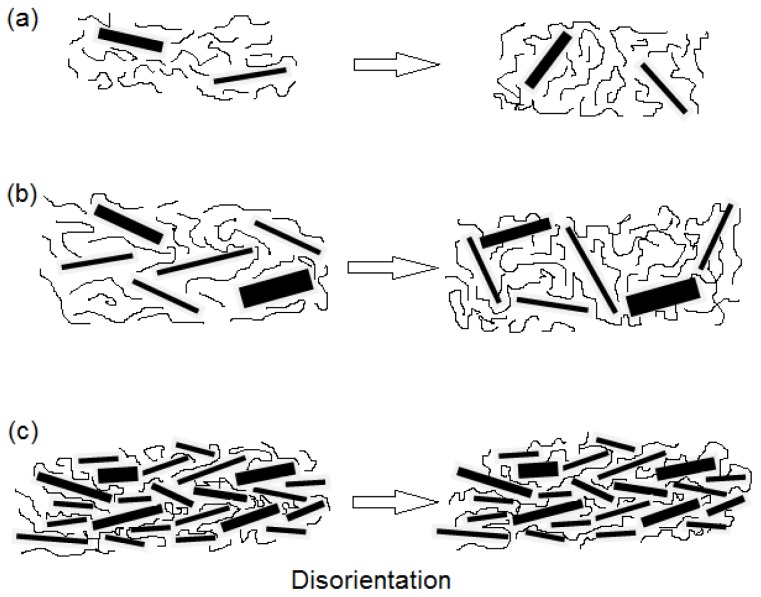
Illustration of disorientation mechanism of xGnP at (**a**) 0.1 wt%; (**b**) 0.3 wt% and (**c**) >0.5 wt%.

Tensile properties of the PLA/EPO/xGnP nanocomposites containing various xGnP contents were examined at room temperature, as shown in Figure 4, which displays stress-strain curves of the nanocomposites. The effect of xGnP loading on tensile strength of PLA composites is depicted in Figure 5. The aim of incorporating xGnP into the polymer matrix is to improve its mechanical properties. The homogeneity of composites, orientation of the reinforcements and the strong interfacial interaction between xGnP and the polymer matrix should have a significant effect on the mechanical properties. Tensile strength of PLA/5EPO increase as xGnP loading increases and attains the highest value (41.07 MPa) at 0.3 wt% xGnP loading. At 0.1 wt% of graphene nanoplatelets loading, the reinforcement effect is limited due to the density of filler is not high enough to form a percolated network. As illustrated in Figure 6a, even complete randomization of the graphene nanoplatelets at very low concentration (0.1 wt%) will not result in graphene contact since their spheres of rotation will not intersect. For 0.3 wt% xGnP loading, the xGnP dispersion and distribution started to improve when the graphene nanoplatelets concentration becomes greater. Therefore, the tensile results imply better strength compared to 0.1 wt% xGnP loading. There is xGnP-xGnP and xGnP-matrix interaction as a result of the percolated network formed (Figure 6b). Further increase of xGnP loading, decreases the tensile strength. In the concentrated regime of xGnP (>0.5 wt%), reorientation cannot be achieved due to excluded volume interactions between nanoplatelets (Figure 6c). When the amount of xGnP reaches a critical content (0.3 wt%) and the distance between two xGnP is so small that they may be apt to stack together easily due to Van der Waals forces [29], thus it decreases in tensile strength.

**Figure 7:**
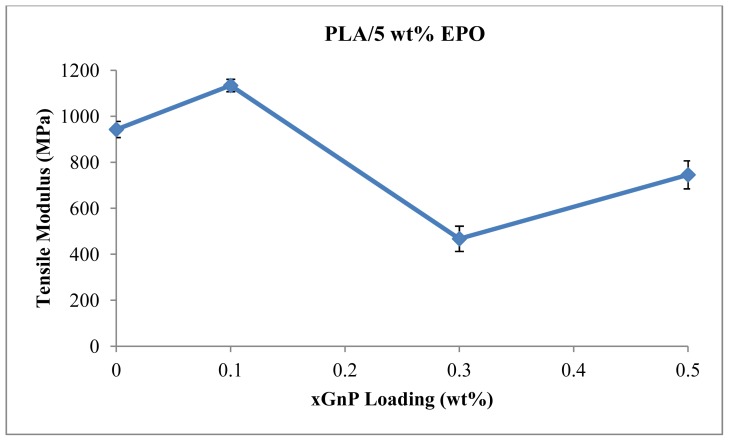
Tensile modulus of PLA/5EPO with various xGnP loadings.

Tensile modulus is a common method to measure the stiffness of a material and is a quantity used to characterize materials [30]. The higher the tensile modulus, the stiffer is the material, thus more stress is required to produce a given amount of strain. Figure 7 shows tensile modulus of PLA/5EPO/xGnP nanocomposites. PLA/5EPO blend exhibited a tensile modulus value of 948 MPa. The addition of 0.3 wt% of xGnP significantly decreases the tensile modulus, and hence the stiffness. This is due to the the fact that nanoplatelets can interact after the rotary relaxation via direct contacts or bridging by polymer chains, and build a sample spanning filler network, which gives rise to the elastic response as illustrated in Figure 6c. This signifies the material is less rigid compared to PLA/5EPO blend. However, at higher xGnP concentration, the close contact between xGnP clusters can give rise to a rigid filler network, which increase the tensile modulus.

**Figure 8:**
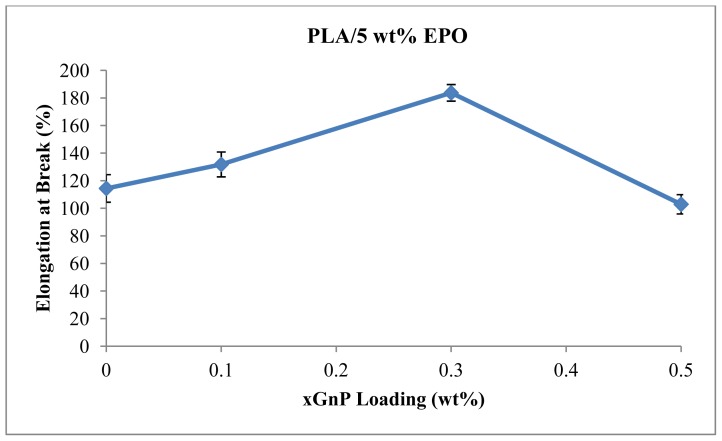
Elongation at break of PLA/5EPO with various xGnP loadings.

Figure 8 shows the elongation at break of PLA/EPO/xGnP composites. PLA/5 wt% EPO blend shows elongation at break value of 114.4 MPa. It increases with the addition of xGnP. The highest elongation at break (183.7%) is observed from PLA/5EPO with 0.3 wt% xGnP loading. Further addition of xGnP causes the decrease of elongation at break which made the blend more brittle. The reason for this may be attributed to a large aspect ratio of the rigid filler and the interaction between xGnP and the matrix, which restricts the movement of the polymer chains [29]. The trend of the elongation at break is the inverse of the tensile modulus.

From the mechanical tensile test, we can conclude that there is presence of a critical amount of xGnP loading on the mechanical properties. The critical amount of xGnP loading is 0.3 wt%. At this critical amount, the xGnP is well dispersed in the polymer matrix and brings about significant improvement to the mechanical properties, while further addition of xGnP may result in stacking of the nanoplatelets, lowering the efficiency of the mechanical improvement.

### 2.4. Flexural Properties

**Figure 9:**
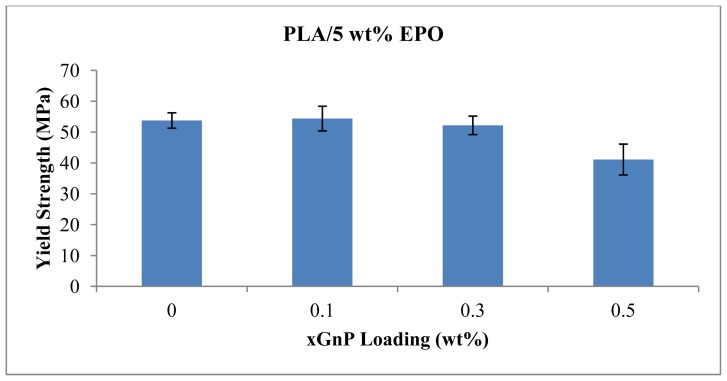
Yield strength of PLA/5EPO with various xGnP loadings.

**Figure 10:**
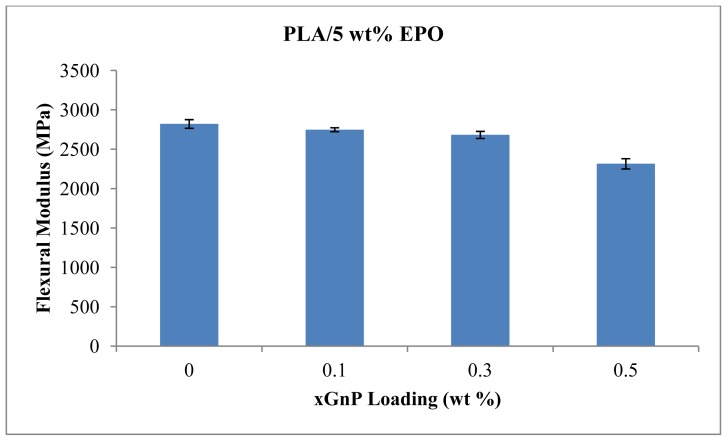
Flexural modulus of PLA/5EPO with various xGnP loadings.

Flexural properties of the composites provide information concerning the load energy applied to break the samples. Our previous study showed that PLA/EPO blend at a weight ratio of 95/5 has the best tensile properties, especially in elongation at break [11]. Therefore this ratio was used in the subsequent experiments. The flexural yield strength and flexural modulus of PLA/5 wt% EPO with various xGnP contents are shown in Figures 9 and 10, respectively.

In general, an increase in xGnP loadings has no significant effect on the flexural strength and modulus. They are characterized by comparable values to those recorded for neat PLA/5 wt% EPO blend at least at low xGnP loading (0.3 wt%). As expected, higher xGnP content (*i.e*., 0.5 wt%) leads to significant decrease in both flexural yield strength and modulus.

### 2.5. Impact Strength

**Figure 11:**
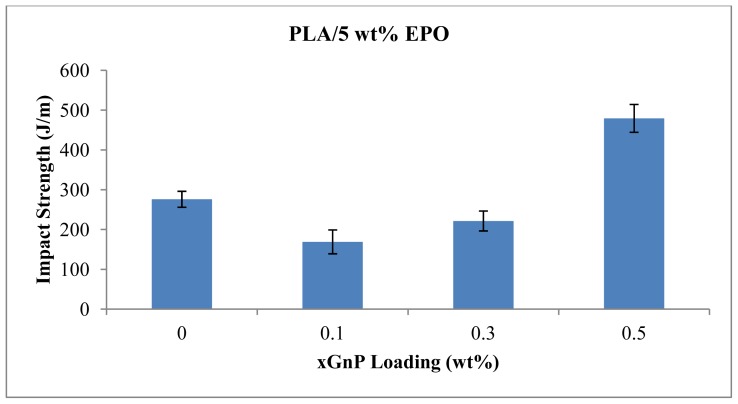
Impact strength of PLA/5 wt% EPO with various xGnP loadings.

Impact test reflects the ability of material absorbing energy at fracture, when exposed to sudden impact. The impact strength of PLA/5 wt% EPO improved by 73.6% with the presence of 0.5 wt% xGnP loading as shown in Figure 11. The impact strength of PLA/5EPO and PLA/5EPO/0.5 wt% xGnP are 276.0 J/m and 479.2 J/m, respectively. It shows around 73.6% increases in impact strength when 0.5 wt% of xGnP was incorporated into PLA/5EPO blend. At lower xGnP content, the impact strength is low. The difference in impact strength may result from the different adhesion between the xGnP sheets and the polymer matrix, as well as the difference in dispersion state in the polymer. Those differences eventually will result in various energy absorbing mechanisms at the impact fracture surface, such as crack branching due to hindrance by reinforcements, bridging of the crack, creation of voids and crazes [31]. However, the adhesion forces between the xGnP and the polymer molecules are of a van der Waals nature, which may not be sufficient magnitude to transfer the stress to xGnP.

### 2.6. Scanning Electron Microscopy

**Figure 12:**
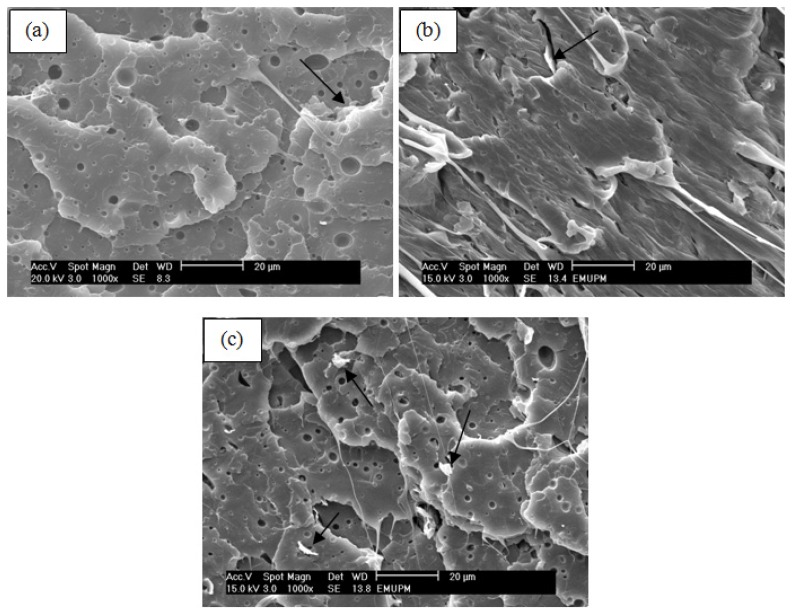
SEM micrographs of PLA/5EPO with (**a**) 0.1 wt% xGnP; (**b**) 0.3 wt% xGnP and (**c**) 0.5 wt% xGnP.

The fracture surface of the nanocomposites was examined by scanning electron microscope to study the morphology of the surface. Figure 12 shows SEM micrographs of fracture surface of PLA/5EPO with various loadings of xGnP at magnification of 1000×. As shown in Figure 12, the dark background represents the PLA polymer matrix while bright areas represent xGnP sheets (as shown by the arrow) distributed in the polymer matrix. The conducting xGnP and the insulating polymer matrix resulted in the contrast between xGnP network and polymer matrix. As can be observed in Figure 12b, the PLA/5EPO/0.3 wt% xGnP nanocomposite is more homogenous and displays good uniformity. Good uniformity of composites indicates good degree of dispersion of the nanofiller and therefore results in better tensile strength as shown in Figure 5. PLA/5EPO/0.3 wt% xGnP also exhibit a strong stretching effect conforming to the high elongation at break during tensile testing. This agrees with the elongation at break result which gives the highest values (183.7%). Notice that in the micrographs, the voids are presented due to EPO dispersed on the PLA matrix (EPO rich phase). The presence of voids results in poor mechanical strength. However, with the addition of 0.3 wt% xGnP to PLA/5EPO, the blend showed good interaction resulting in higher mechanical properties. Nanocomposite with 0.1 and 0.5 wt% did not show improvement in the interaction, as can be observed through SEM micrographs.

## 3. Experimental Section

### 3.1. Materials

**Figure 13:**
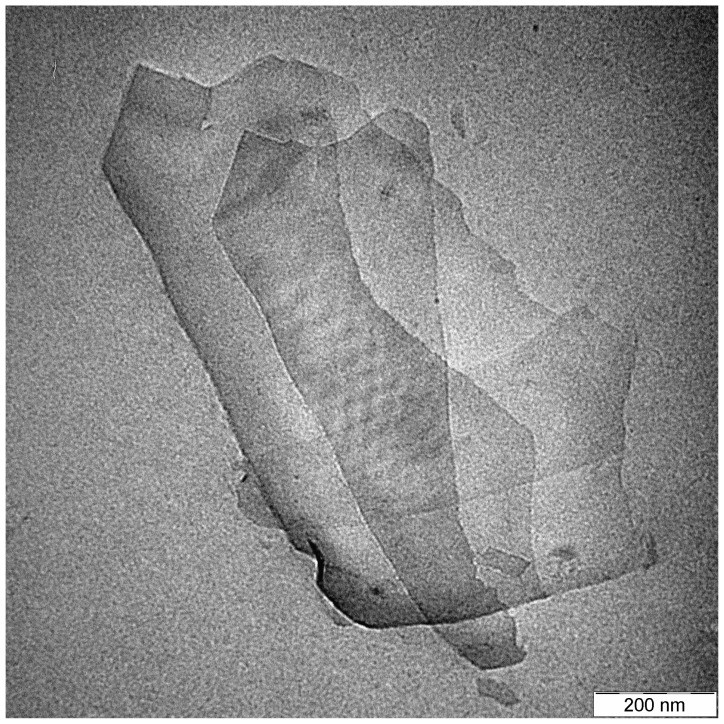
TEM micrograph of graphene nanoplatelets.

Poly(lactic acid) resin, commercial grade 4042D, Mw ~ 390,000 Da, was supplied by NatureWorks^®^ LCC, Minnesota USA. Epoxidized palm oil (EPO) was supplied by Malaysian Palm Oil Board (MPOB, Malaysia). The characteristics of the EPO obtained are listed in Table 1. Graphene nanoplatelets, trade name xGnP^®^, was supplied by XG sciences Inc, Michigan. Each particle consists of several sheet of graphene with an average thickness of approximately 6–8 nanometers, average diameter of 15 microns. Figure 13 is a micrograph of graphene nanoplatelets attained by transmission electron microscope.

### 3.2. Preparation of PLA/EPO/Graphene Nanoplatelets Nanocomposites

The PLA/EPO/xGnP nanocomposites were melt blended by Thermo Haake Polydrive internal mixer with 50 rpm of the rotor speed, at 170 °C for 15 min. EPO was used as plasticizer to PLA. The weight ratio of PLA to EPOwas kept constant at 95/5. The xGnP content was varied between 0.1 wt% to 0.5 wt%. The composites obtained were then molded into sheets of 1 mm in thickness by hot pressing at 165 °C for 10 min at the pressure of 110 kg/cm^2^, followed by cooling to room temperature. The sheets were used for further characterization.

### 3.3. Characterization

#### 3.3.1. Tensile Properties Measurement

Tensile properties test were carried out by using Instron 4302 series IX. The samples were cut into dumbbell shape following ASTM D638 (type V) standard. Load of 1.0 kN was applied at constant crosshead speed of 10 mm/min at room temperature. Tensile strength, tensile modulus and elongation at break were evaluated from the stress-strain data. Five experimental data were used in the computation. The error bars signifies standard deviation of the data obtained from each analysis.

#### 3.3.2. Flexural Test

Flexural test was conducted in accordance with ASTM D790, using Instron Universal Testing Machine (Model 4302 Series IX) equipped with a 1 kN load cell. Seven specimens in rectangular shape with the dimension 127.00 mm × 12.70 mm × 3.00 mm of size were tested for each composition. Yield strength and flexural modulus were obtained at constant crosshead speed of 3 mm/min. An average of five results was taken as the resultant value. The error bars signifies standard deviation of the data obtained from each analysis.

#### 3.3.3. Izod Impact Test

Izod Impact test was based on ASTM D256 standard. The dimension of the specimen was 63.5 mm (length) × 12.7 mm (width) × 3.0 mm (thickness). The specimens were held as vertical cantilever beam and were impacted on the notched face by a single swing of the pendulum. The work-of-fracture values were calculated by dividing the energy obtained with the thickness of the specimens.

#### 3.3.4. X-ray Diffraction (XRD)

X-ray diffraction measurement was carried out by using a Shimadzu XRD 6000 X-ray diffractometer with CuK_α_ radiation (λ = 1.542 Å) operated at 30 kV and 30 mA. Data were recorded in 2*θ* range of 2°–10° at the scan rate of 2°/min.

#### 3.3.5. Brunauer–Emmet–Teller (BET)

BET specific surface area was determined from nitrogen adsorption by using a Quantochrome surface area analyzer model Autosorb-1 at liquid nitrogen temperature.

#### 3.3.6. Scanning Electron Microscopy (SEM)

The scanning electron micrographs of tensile fracture surface of the samples were recorded by a Philips XL30 ESEM scanning electron microscope operated at 20 kV. The samples were coated with gold by a Bio-rad coating system before viewing. The scanning electron micrographs were recorded at magnification of 1000×.

## 4. Conclusions

PLA/EPO/xGnP green nanocomposites were successfully prepared by the melt blending method. The prepared nanocomposites exhibited a significant improvement in mechanical properties at a low xGnP loading. At 0.3 wt% xGnP loading, the tensile strength and elongation at break attained maximum values, with an increase of 26.5% and 60.6%, respectively. The enhancement to some extent of the mechanical properties of the PLA/EPO/xGnP nanocomposites can be ascribed to the homogeneous dispersion and orientation of the xGnP nanoplatelets in the polymer matrix and strong interfacial interactions between both components. SEM results prove the enhancement of tensile strength and elongation at break at 0.3 wt% xGnP loading.

## Figures and Tables

**Table 1:** Properties of EPO.

Sample Composition	Epoxidized palm olein
Oxygen Oxirane Content (%)	3.2309
Acid Value (mg KOH/g sample)	0.4287
Iodine Value (g I_2_/100 g sample)	0.6371
Moisture Content	0.08
pH	5–6
